# Longitudinal Hepatic and Metabolic Trajectories Following Dolutegravir Initiation in Antiretroviral Therapy (ART)-Naïve People Living With HIV: A Prospective Cohort Study From South India

**DOI:** 10.7759/cureus.112448

**Published:** 2026-07-11

**Authors:** Savitha A Sebastian, Jose Joseph, Sumithra Selvam

**Affiliations:** 1 Internal Medicine, St. John's Medical College Hospital, Bangalore, IND; 2 Critical Care Medicine, St. John's Medical College Hospital, Bangalore, IND; 3 Biostatistics, St. John's Research Institute, Bangalore, IND

**Keywords:** antiretroviral therapies, dolutegravir, drug-induced liver injury (dili), hepatic adaptation, hiv aids, india, liver enzyme elevation, metabolic abnormalities

## Abstract

Background

Dolutegravir (DTG)-based combination antiretroviral therapy (cART), specifically the tenofovir disoproxil fumarate, lamivudine, and dolutegravir (TLD) regimen, is being widely prescribed for the management of people living with HIV (PLHIV) in India and other low- and middle-income countries. Although DTG is well-tolerated, concerns persist regarding its hepatic and metabolic safety. Prospective real-world data characterizing the longitudinal hepatic and metabolic trajectories following DTG initiation in antiretroviral therapy (ART)-naïve South Asian PLHIV remain limited. Therefore, we conducted a prospective study to determine the incidence and grading of liver enzyme elevation (LEE) and characterize the longitudinal trajectories of alanine aminotransferase (ALT), anthropometric, and glycemic parameters over six months in ART-naïve PLHIV initiated on the TLD regimen.

Methodology

We conducted a six-month prospective cohort study and followed 101 ART-naïve adult PLHIV initiated on the TLD regimen at a tertiary care hospital in Bengaluru, India. Serum ALT, weight, body mass index (BMI), waist circumference, and random blood sugar (RBS) were measured at baseline and at one, two, three, and six months. Repeated-measures analysis of variance (rmANOVA) was used to assess longitudinal trends; paired-samples t-test compared baseline and six-month values for waist circumference; chi-square tests examined categorical associations.

Results

All 101 enrolled participants completed the six-month follow-up. The mean age was 36.5 ± 11.2 years, and 67 (66.3%) participants were male. Any-grade LEE occurred in 22 (21.8%) participants. Mean ALT rose transiently from 26 ± 14 IU/L at baseline to a peak of 32 IU/L at one month, then declined progressively to 27 ± 17 IU/L at six months; the overall trajectory was not statistically significant (*F*(1.94, 192.29) = 1.114, *P* = 0.329). All participants with LEE remained asymptomatic; no jaundice, coagulopathy, or hepatic encephalopathy was observed in this cohort. Statistically significant increases were noted in weight +1.79 kg (*F*(2.02, 201.87) = 12.549, *P* < 0.001), and mean BMI +0.71 kg/m² (*F*(2.00, 199.90) = 13.117, *P* < 0.001). Mean RBS rose by only +1.06 mg/dL (*F*(3.71, 370.96) = 2.274, *P* = 0.066) and waist circumference by +0.17 cm (*t*(100) = 1.42, *P* = 0.159) - neither change reached statistical significance. LEE was not significantly associated with age (*t*(99) = 0.298, *P* = 0.766), sex (χ²(1) = 3.02, *P* = 0.082, φ = 0.17), concurrent anti-tuberculosis therapy (Fisher's exact *P* = 0.116), or alcohol use (χ²(1) = 0.001, *P* = 0.973, φ = 0.003).

Conclusions

In ART-naïve South Indian PLHIV initiating DTG-based cART, LEEs were relatively common but were predominantly mild, asymptomatic, and self-limited. LEE was characterized by an early rise in ALT followed by a spontaneous decline in most cases by six months despite uninterrupted therapy, a pattern that may reflect hepatic adaptation. In contrast, body weight and BMI increased progressively throughout follow-up, suggesting that hepatic and metabolic responses to DTG may follow distinct trajectories. Overall, ALT elevations were predominantly mild and self-limited, indicating favorable hepatic tolerability of DTG-based cART in this cohort while highlighting the need for continued metabolic monitoring.

## Introduction

Integrase strand transfer inhibitors (INSTIs), in particular dolutegravir (DTG), are integral components of contemporary antiretroviral therapy (ART), owing to their potent virological efficacy, high genetic barrier to resistance, and favorable tolerability profile [1]. The fixed-dose combination of tenofovir disoproxil fumarate (TDF), lamivudine (3TC), and DTG (TLD) is the World Health Organization's preferred first-line regimen. It has been adopted across national programs throughout Asia and sub-Saharan Africa, the regions that bear most of the global HIV burden [2].

Serum liver enzyme elevations (LEEs) have been documented with all major classes of antiretroviral agents: nucleoside/nucleotide reverse transcriptase inhibitors (NRTIs), non-nucleoside reverse transcriptase inhibitors (NNRTIs), and protease inhibitors (PIs). In this context, large randomized trials and network/meta‑analyses have shown that DTG has similar or better overall hepatic safety than first‑generation NNRTIs and boosted PIs, with fewer drug‑related discontinuations and no excess of serious hepatic events reported [3,4]. Nevertheless, case reports of treatment‑emergent hepatotoxicity, including sub‑acute liver failure, and post‑marketing pharmacovigilance signals for hepatobiliary disorders and drug‑induced liver injury (DILI) have raised concerns about the hepatic safety profile of DTG [5-7].

At the same time, metabolic dysfunction associated with DTG has been recognized, leading to greater weight gain than older regimens, together with emerging signals of dysglycemia and dyslipidemia [8]. For example, in a large North American cohort study of treatment-naïve people living with HIV (PLHIV), Bourgi et al. reported a mean weight gain of approximately 6 kg over 18 months following DTG initiation - significantly greater than that observed on alternative first-line regimens [9]. Emerging evidence also suggests an increased risk of hyperglycemia, with Lartey et al. reporting incident type 2 diabetes in 11.8% of PLHIV within 72 weeks of DTG initiation [10]. Likewise, in the large prospective RESPOND cohort, dyslipidemia occurred at a rate of 191.6 per 1,000 person-years, with individuals receiving DTG-based therapy experiencing a higher risk than those treated with NNRTI-based regimens [11]. These observations have raised concerns regarding the broader metabolic consequences of DTG-based treatment, particularly in populations already at elevated long-term cardiometabolic risk.

Emerging Indian data have generally supported DTG's favorable safety profile while highlighting important metabolic and hepatic signals. Real-world and phase IV studies have reported low rates of clinically significant hepatotoxicity, with LEEs occurring in approximately 15% of patients and most resolving spontaneously, alongside mean weight gains of 2-4 kg within 24-96 weeks and occasional reports of treatment-emergent hyperglycemia [12-15]. However, these studies have largely focused on discrete clinical outcomes or assessments at selected time points, leaving the temporal evolution of hepatic and metabolic changes following DTG initiation incompletely characterized, particularly in treatment-naïve Indian PLHIV.

To our knowledge, Indian data on prospective hepatic and metabolic trajectories within a single ART-naïve cohort receiving TDF-3TC-DTG (TLD) are unavailable. Characterizing these trajectories will improve understanding of DTG-associated physiological adaptation, inform laboratory monitoring, and help distinguish transient abnormalities from those requiring further evaluation or treatment modification. We therefore conducted a prospective study over six months to determine the incidence of LEE and characterize longitudinal serum alanine aminotransferase (ALT) trends alongside body weight, body mass index (BMI), and blood sugars among ART-naïve PLHIV initiated on DTG-based combination antiretroviral therapy (cART) at a tertiary care center in Bengaluru, India.

## Materials and methods

Study design and setting

This prospective cohort study was conducted at the HIV clinic of St. John's Medical College Hospital (SJMCH), Bengaluru, a city in southern India, between August 2022 and August 2024. SJMCH is a tertiary care medical college, and the clinic primarily serves a lower socioeconomic population, with ART available under the government-funded national program. The study was approved by the Institutional Ethics Committee of SJMCH (Ref. No. ST-179/2022), and all participants were enrolled after providing written informed consent. The follow-up period was six months with five time points of observation: baseline, one, two, three, and six months.

Participants and eligibility criteria

Adult PLHIV (age ≥18 years) who were ART-naïve and initiated on the tenofovir disoproxil fumarate (TDF) 300 mg, lamivudine (3TC) 300 mg, and DTG 50 mg (TLD) regimen were enrolled using consecutive sampling. Pregnant women were excluded from the cohort. Patients with diabetes, pre-existing liver disease, hepatitis B or C co-infection, or concurrent use of potentially hepatotoxic medications, including anti-tuberculosis therapy (ATT), were not formally excluded. The eligibility criteria applied during enrollment are summarized in Table 1.

**Table 1:** Eligibility criteria for study enrollment. ART, antiretroviral therapy; TLD, tenofovir disoproxil fumarate-lamivudine-dolutegravir; TDF, tenofovir disoproxil fumarate; 3TC, lamivudine; DTG, dolutegravir

Inclusion criteria	Exclusion criteria
Age ≥18 years	Pregnancy
HIV infection	
Initiated on tenofovir disoproxil fumarate-lamivudine-dolutegravir (TLD) regimen (TDF 300 mg, 3TC 300 mg, DTG 50 mg)	
ART-naïve before initiation of TLD	

Data collection and follow-up

A standardized proforma captured baseline sociodemographic data, comorbidities, drug history, and substance use, including smoking and alcohol use. Concurrent use of potentially hepatotoxic drugs was specifically noted, including ATT, statins, non-steroidal anti-inflammatory drugs (NSAIDs), antibiotics, antifungals, antiseizure medication, and complementary and alternative medication (CAM). Alcohol use was recorded as current, past, or never; smoking was recorded as current or ever. Height was measured at baseline; waist circumference at baseline and six months; weight, random blood sugar (RBS), and serum alanine aminotransferase (ALT) were measured at baseline, one, two, three, and six months.

Measurements and laboratory analysis

Anthropometric measurements included height, weight, BMI, and waist circumference. We ensured that all participants were barefoot and wore light clothing before measurements. Height was measured using a stadiometer (Seca, Germany) with participants in the Frankfurt plane with the heels, buttocks, upper back, and head in contact with the stadiometer; height was recorded to the nearest 0.5 cm. Body weight was measured using a calibrated digital weighing scale (Omron, India) and recorded to the nearest 0.1 kg. Waist circumference was measured directly over the skin using a non-stretchable measuring tape (Seca, Germany) at the midpoint between the lower margin of the last palpable rib and the iliac crest, at the end of a normal expiration, and recorded to the nearest 0.5 cm. Blood investigations were performed at the National Accreditation Board for Testing and Calibration Laboratories (NABL)-accredited laboratory of St. John's Medical College Hospital, Bengaluru. Serum glucose was measured using the hexokinase/glucose-6-phosphate dehydrogenase (G6PDH) method. Serum ALT was measured using an enzymatic kinetic assay based on nicotinamide adenine dinucleotide (NADH) consumption, following the International Federation of Clinical Chemistry and Laboratory Medicine (IFCC)-recommended method without pyridoxal-5-phosphate activation. All biochemical analyses were performed on the Abbott Architect platform (Abbott Laboratories, Abbott Park, IL).

Outcomes and variables

The primary outcome was incident liver enzyme elevation (LEE), defined as ALT > 1.25 × upper limit of normal (ULN) consistent with Grade 1 (mild) elevation per the Division of AIDS (DAIDS) grading of severity of adverse events [16]. Secondary outcomes were the longitudinal trajectories of ALT, body weight, BMI, waist circumference, and RBS over the six-month observation period.

The independent variables were age, sex, alcohol use, concomitant use of potentially hepatotoxic drugs, liver disease, and the change from baseline in waist circumference, weight, BMI, and RBS.

ALT grade was classified as: Normal (<1.25 × ULN); Grade 1 (1.25 to <2.5 × ULN; mild); Grade 2 (2.5 to <5.0 × ULN; moderate); Grade 3 (5.0 to <10.0 × ULN; severe); Grade 4 (>10.0 × ULN; potentially life-threatening). The ULN for ALT elevations was defined as 34 IU/L using the institutional laboratory cut-offs for both males and females, which served as the reference standard in routine clinical care throughout the study period. We routinely performed ALT for hepatic monitoring, as recommended by national and other low- and middle-income country (LMIC) guidelines [17,18]. A complete liver function test (LFT) was performed if patients were symptomatic for LEE, had clinical jaundice, or had Grade 3 LEE.

BMI was categorized as "underweight" (<18.5 kg/m²), "normal" (18.5-22.9 kg/m²), "overweight at risk" (23.0-24.9 kg/m²), "obese class I" (25.0-29.9 kg/m²), and "obese class II" (≥30.0 kg/m²) per WHO Asia-Pacific criteria [19].

Hyperglycemia was defined as an RBS value >140 mg/dL. This threshold was selected to provide adequate specificity for clinically relevant dysglycemia in the context of random (non-fasting) measurements. Somannavar et al. demonstrated in an Indian community-based screening study that a random capillary blood glucose value of 140 mg/dL provided optimal sensitivity and specificity for detecting diabetes defined by a two-hour plasma glucose ≥200 mg/dL [20]. Diabetes mellitus was defined per the American Diabetes Association criteria, including a fasting plasma glucose ≥126 mg/dL, a two-hour postprandial plasma glucose ≥200 mg/dL, or a random plasma glucose ≥200 mg/dL in the presence of classic symptoms of hyperglycemia [21].

Sample size estimation

Sample size was estimated using nMaster version 2.0 (Department of Biostatistics, Christian Medical College, Vellore, Tamil Nadu, India) with the single-proportion formula. The OPERA cohort - a large, real-world, North American observational cohort of 16,024 PLHIV, including 6,102 on DTG - provided the best available DTG-specific safety data at the time of study planning [22]. Based on their reported prevalence of approximately 7% for moderate-to-severe ALT elevations, a minimum sample size of 100 participants was calculated assuming a 95% confidence level with 5% absolute precision. Although the present study also evaluated mild ALT elevations, reliable estimates of all-grade ALT elevations were unavailable at the time of protocol development.

Statistical analysis

Descriptive statistics were reported as mean ± standard deviation for continuous variables and as number (percentage) for categorical variables. A repeated-measures analysis of variance (rmANOVA) was used to assess longitudinal trends in mean ALT, weight, BMI, and RBS across study time points. Mauchly's test of sphericity was applied to each rmANOVA, and the Greenhouse-Geisser correction was applied when sphericity was violated. Tests of within-subjects linear contrasts were performed for each parameter to characterize the magnitude and direction of any linear progression over time. The Bonferroni post hoc test was used for pairwise comparison to compare the means between two time points. A complementary rmANOVA on natural log-transformed ALT was performed as a sensitivity analysis to address the right-skewed distribution of ALT at one month. Paired-samples t-tests compared baseline and six-month values for each parameter. Chi-square and Fisher's exact tests examined associations between LEE and categorical risk factors (sex, alcohol use, ATT co-administration, hepatitis co-infection). Mann-Whitney U tests were used to compare changes in anthropometric parameters between participants with and without LEE. A two-sided *P*-value <0.05 was considered statistically significant. Data were entered into Microsoft Excel and analyzed using IBM SPSS Statistics version 29.0 (IBM Corp., Armonk, NY).

## Results

Baseline characteristics

We enrolled 101 ART-naïve PLHIV, initiating DTG-based cART, with none lost to follow-up. The mean age of the participants was 36.5 ± 11.2 years, and two-thirds were male (*n* = 67, 66.3%). At baseline, the cohort had a mean weight of 61.0 ± 13.5 kg, a mean BMI of 22.6 ± 4.5 kg/m², and a mean waist circumference of 92.7 ± 8.3 cm; mean baseline RBS was 93.7 ± 15.0 mg/dL.

Comorbidities of concern included active tuberculosis on ATT in 6 (5.9%) participants and chronic hepatitis B in 1 (1.0%) participant; 2 (1.9%) participants each reported hypertension and hypothyroidism. A total of 4 (3.9%) participants reported current alcohol use, and 5 (4.9%) participants reported tobacco use. The baseline characteristics of the cohort are summarized in Table 2.

**Table 2:** Baseline characteristics of the cohort (n = 101). SD, standard deviation; ALT, alanine aminotransferase; BMI, body mass index; WC, waist circumference; RBS, random blood sugar; ATT, anti-tuberculosis therapy

Characteristic	Value	Unit
Age (mean ± SD)	36.5 ± 11.2	Years
Sex		
Male	67 (66.3)	*n* (%)
Female	34 (33.7)	*n* (%)
Baseline ALT (mean ± SD)	26.0 ± 14.4	IU/L
Baseline weight (mean ± SD)	61.0 ± 13.5	kg
Baseline BMI (mean ± SD)	22.6 ± 4.5	kg/m²
Baseline WC (mean ± SD)	92.74 ± 8.3	cm
Baseline RBS (mean ± SD)	93.7 ± 15.0	mg/dL
Comorbidities		
Active tuberculosis on ATT	6 (5.9)	*n* (%)
Hepatitis B	1 (1.0)	*n* (%)
Hypertension	2 (1.9)	*n* (%)
Hypothyroidism	2 (1.9)	*n* (%)
Alcohol use		
Current	4 (3.9)	*n* (%)
Past	5 (4.9)	*n* (%)
Tobacco use		
Current	5 (4.9)	*n* (%)
Ever used	2 (1.9)	*n* (%)

Incidence and grading of LEE

During the six-month follow-up, of the 101 participants, 22 (21.78%) developed any-grade incident LEE (Grade 1 or higher; ALT >1.25 × ULN). Clinically significant LEE (Grade 2 or higher; ALT >2.5 × ULN) occurred in 7 (6.93%) participants - 5 (5%) had Grade 2 (moderate) elevations, 1 (1.0%) had Grade 3 (severe), and 1 (1.0%) had Grade 4 (potentially life-threatening). No participant developed clinical features of jaundice or acute hepatic failure. The participant with Grade 4 LEE recorded an ALT of 405 IU/L at one month. He was asymptomatic, and a reflex liver function test revealed normal bilirubin levels, an aspartate aminotransferase (AST) of 283 IU/L, and a mild alkaline phosphatase (ALP) elevation at 164 IU/L. In this case, the medical team would typically have temporarily stopped ART. However, the medical team was unable to contact the participant, and therefore, ART interruption was not possible. Despite this, the elevation declined within one month, despite the uninterrupted continuation of DTG-based ART. The distribution of ALT grades at each observation point is presented in Table 3.

**Table 3:** Distribution of ALT grades over time among PLHIV initiated on DTG-based cART (n = 101). This table summarizes the distribution of ALT grades at baseline and during follow-up (one, two, three, and six months) among PLHIV initiated on DTG-based cART. Grade 1 (mild): 1.25 to <2.5 × ULN; Grade 2 (moderate): 2.5 to <5.0 × ULN; Grade 3 (severe): 5.0 to <10.0 × ULN; Grade 4 (potentially life-threatening): >10.0 × ULN. ULN for ALT = 34 IU/L. Data are presented as n (%). Percentages were calculated using the total study population (n = 101) as the denominator. PLHIV, people living with HIV; DTG, dolutegravir; cART, combination antiretroviral therapy; ALT, alanine aminotransferase; ULN, upper limit of normal

ALT grade	Baseline, *n* (%)	One month, *n* (%)	Two months, *n* (%)	Three months, *n* (%)	Six months, *n* (%)
Normal	90 (89.1)	87 (86.1)	90 (89.1)	91 (90.1)	92 (91.0)
Grade 1 (Mild)	11 (10.9)	10 (9.9)	8 (7.9)	8 (7.9)	8 (7.9)
Grade 2 (Moderate)	0 (0.0)	2 (2.0)	2 (2.0)	2 (2.0)	1 (1.0)
Grade 3 (Severe)	0 (0.0)	0 (0.0)	1 (1.0)	0 (0.0)	0 (0.0)
Grade 4 (Potentially life-threatening)	0 (0.0)	1 (1.0)	0 (0.0)	0 (0.0)	0 (0.0)

Longitudinal liver enzyme trends

**Figure 1:**
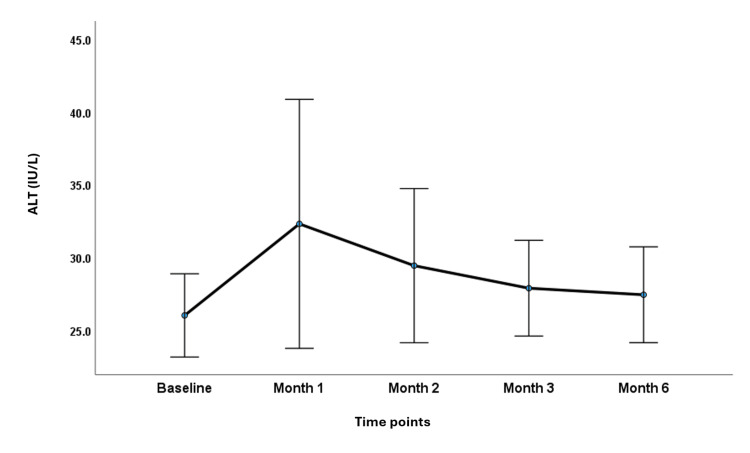
Mean serum ALT trajectory over six months of the HIV cohort initiated on DTG-based antiretroviral therapy (n = 101). Mean serum ALT at baseline and one, two, three, and six months following dolutegravir-based combination antiretroviral therapy initiation in ART-naïve people living with HIV (*n* = 101). Data points represent the mean ALT concentration at each time point, with vertical bars indicating the 95% confidence interval. The cohort-level mean rose transiently from 26.0 IU/L at baseline to a peak of 32.3 IU/L at one month, then declined progressively to 27.4 IU/L by six months. Repeated-measures ANOVA with Greenhouse-Geisser correction demonstrated no statistically significant change in mean ALT across the five time points (*F*(1.94, 192.29) = 1.114, *P* = 0.329). The trajectory is illustrative of a transient early peak followed by spontaneous normalization despite uninterrupted drug continuation. DTG, dolutegravir; ART, antiretroviral therapy; ALT, alanine aminotransferase; ANOVA, analysis of variance

We examined the longitudinal trajectory of serum ALT across all five observation time points. Mean ALT rose transiently from a baseline of 25.99 ± 14.42 IU/L to a peak of 32.28 ± 43.11 IU/L at one month, then declined progressively to 29.41 ± 26.69 IU/L at two months, 27.86 ± 16.58 IU/L at three months, and 27.41 ± 16.60 IU/L at six months. rmANOVA, with Greenhouse-Geisser correction, demonstrated no statistically significant change in mean ALT across the five time points (*F*(1.94, 192.29) = 1.114, *P* = 0.329), and the within-subjects linear contrast was also non-significant (*F*(1, 99) = 0.18, *P* = 0.67), indicating no significant linear progression in ALT over the observation period (Table 4). To address the right-skewed distribution of ALT at one month, a complementary rmANOVA on natural log-transformed ALT was performed as a sensitivity analysis and yielded a comparable non-significant result (*F*(3.13, 310.21) = 0.847, *P* = 0.473), confirming the robustness of this finding. The longitudinal trajectory is depicted in Figure 1 and shows a characteristic transient rise peaking at one month, followed by gradual normalization over the subsequent observation time points.

**Table 4:** Mean values of ALT, weight, BMI, and RBS at all observation time points (n = 101). Values are presented as mean ± standard deviation (SD). The Δ (6 months) column shows the mean increase from baseline to six months. Repeated-measures analysis of variance (ANOVA) was used to assess longitudinal trends across five observation time points. The Greenhouse-Geisser correction was applied to all four parameters, as Mauchly's test indicated a violation of sphericity. ALT, alanine aminotransferase; BMI, body mass index; RBS, random blood sugar

Parameter	Baseline mean (SD)	1 month, mean (SD)	2 months, mean (SD)	3 months, mean (SD)	6 months, mean (SD)	Δ (6 months)	Test statistic	*P*-value
ALT (IU/L)	25.99 ± 14.42	32.28 ± 43.11	29.41 ± 26.69	27.86 ± 16.58	27.41 ± 16.60	+1.4	*F*(1.94, 192.29) = 1.114	0.329
Weight (kg)	61.01 ± 13.49	61.29 ± 12.89	61.93 ± 12.52	62.41 ± 12.72	62.80 ± 12.72	+1.79	*F*(2.02, 201.87) = 12.549	<0.001
BMI (kg/m²)	22.61 ± 4.49	22.72 ± 4.30	22.97 ± 4.17	23.15 ± 4.30	23.32 ± 4.36	+0.71	*F*(2.00, 199.90) = 13.117	<0.001
RBS (mg/dL)	93.66 ± 15.02	94.57 ± 18.72	98.36 ± 16.53	97.30 ± 17.48	94.72 ± 16.16	+1.06	*F*(3.71, 370.96) = 2.274	0.066

**Figure 2:**
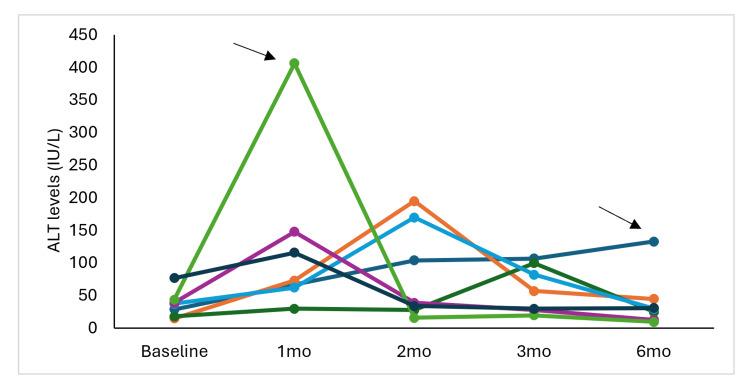
ALT trajectories in individuals with Grade 2 or higher liver enzyme elevations on DTG-based ART (n = 7). This figure depicts the individual serum ALT trajectories of the seven participants who developed clinically significant liver enzyme elevation (peak ALT ≥2.5 × upper limit of normal; Grade 2 or higher) following initiation on DTG-based combination ART. Each colored line represents one participant's ALT measurements at baseline and at one, two, three, and six months; the horizontal axis indicates the observation time point, and the vertical axis shows ALT concentration in IU/L. Despite peak values reaching 405 IU/L (>10 × ULN; Grade 4), six of the seven participants demonstrated the same temporal pattern: an early peak at one to two months followed by spontaneous decline toward baseline by six months, despite ART continuation, consistent with hepatic adaptation. One participant followed a distinct trajectory with progressively rising ALT through six months, suggesting a non-adaptive response in this individual. No participant required ART discontinuation or developed clinical jaundice, coagulopathy, or other features of decompensated liver injury. Arrows highlight the participant with the highest ALT elevation (405 IU/L at one month) and the participant with persistent ALT elevation during follow-up. Image generated by authors using Microsoft Excel (Microsoft Corporation, Redmond, WA). ALT, alanine aminotransferase; DTG, dolutegravir; ULN, upper limit of normal; ART, antiretroviral therapy

This characteristic pattern of an early transient ALT rise followed by decline was particularly pronounced among a subgroup of 7 (6.93%) participants with LEE Grade 2 or higher. Among the seven participants, six demonstrated the same temporal pattern of early peak at one to two months followed by spontaneous decline, returning to values at or near the ULN by three and six months despite uninterrupted continuation of DTG-based cART. One participant followed a distinct trajectory, with progressively rising ALT levels over six months. No participant required ART discontinuation or developed clinical jaundice, coagulopathy, or other features of decompensated liver injury (Figure 2).

Weight, BMI, and blood sugar

Body weight and BMI increased progressively over the six-month observation period, with the increases being statistically significant. Mean weight rose from 61.01 ± 13.49 kg at baseline to 62.80 ± 12.72 kg at six months - a mean increase of 1.79 kg (*F*(2.02, 201.87) = 12.549, *P* < 0.001). Mean BMI increased in parallel from 22.61 ± 4.49 kg/m² at baseline to 23.32 ± 4.36 kg/m² at six months, representing a mean increase of 0.71 kg/m² (*F*(2.00, 199.90) = 13.117, *P* < 0.001). This increase shifted the cohort mean from the upper end of the WHO Asia-Pacific normal BMI range to the *overweight-at-risk* category (23.0-24.9 kg/m²). Bonferroni-adjusted pairwise comparisons confirmed that weight and BMI at months three and six were significantly higher than at baseline. Tests of within-subjects linear contrasts demonstrated highly significant linear trends for both anthropometric parameters (BMI: *F*(1, 100) = 19.21, *P* < 0.001; weight: *F*(1,100) = 17.97, *P* < 0.001), indicating steady monotonic increases rather than transient shifts. Mean waist circumference did not change significantly over six months (+0.17 cm; 92.74 ± 8.28 → 92.91 ± 8.14 cm; *t*(100) = 1.42, *P* = 0.159).

**Figure 3:**
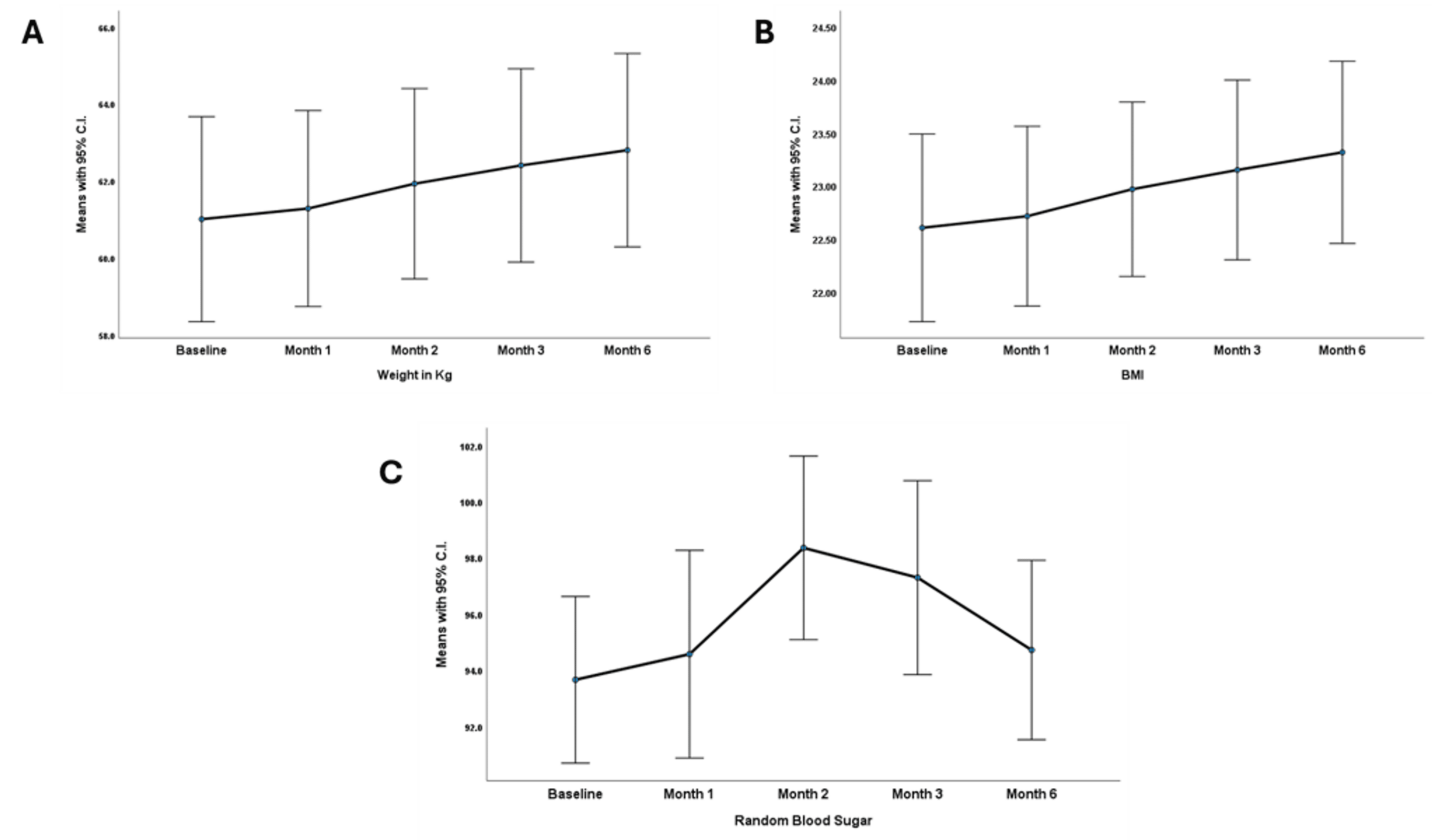
Longitudinal trajectories of mean weight, BMI, and RBS after initiation of dolutegravir-based ART (n = 101). Mean weight, BMI, and RBS at baseline and one, two, three, and six months following dolutegravir-based combination ART initiation in ART-naïve people living with HIV (*n* = 101). Data points represent the mean value at each observation time point, with vertical bars indicating the 95% confidence interval. Panel A (top left) shows mean weight increasing progressively from 61.0 kg at baseline to 62.8 kg at six months (rmANOVA *F*(2.02, 201.87) = 12.549, *P* < 0.001). Panel B (top right) shows mean BMI increasing in parallel from 22.6 kg/m² at baseline to 23.3 kg/m² at six months (rmANOVA *F*(2.00, 199.90) = 13.117, *P* < 0.001). Panel C (bottom) shows mean RBS, which increased transiently from 93.7 mg/dL at baseline to a mid-period peak of 98.4 mg/dL at two months before declining to 94.7 mg/dL at six months (rmANOVA *F*(3.71, 370.96) = 2.274, *P* = 0.066). The Greenhouse-Geisser correction was applied to all three parameters, as Mauchly's test indicated a violation of sphericity. BMI, body mass index; RBS, random blood sugar; rmANOVA, repeated-measures analysis of variance

In contrast, mean RBS showed only a transient mid-period rise (baseline 93.66 ± 15.02 → 98.36 ± 16.53 at two months → 94.72 ± 16.16 at six months) - a net six-month increase of only +1.06 mg/dL. The overall trend approached but did not reach statistical significance (*F*(3.71, 370.96) = 2.274, *P* = 0.066), and the linear contrast was also non-significant (*F*(1, 100) = 1.17, *P* = 0.28). Mean values, mean changes from baseline, and corresponding test statistics for weight, BMI, and RBS are summarized in Table 4. The longitudinal trajectories of these three parameters are illustrated in Figures 3A-3C.

Risk factor analysis

Univariate analysis revealed no significant baseline predictors of incident LEE. Mean age did not differ between participants with and without LEE (35.9 ± 10.4 vs. 36.7 ± 11.5 years; *t*(99) = -0.298, *P* = 0.766). LEE occurred in 18 (26.9%) of 67 males and 4 (11.8%) of 34 females, a difference that did not reach conventional significance (χ²(1) = 3.02, *P* = 0.082, φ = 0.17). Concurrent ATT was associated with a numerically higher incidence of LEE (3/6, 50.0%, vs. 19/95, 20.0%), but this difference did not reach statistical significance (Fisher's exact *P* = 0.116). Alcohol use showed no association with LEE (χ²(1) = 0.001, *P* = 0.97, φ = 0.003). The single hepatitis B-positive participant did not develop clinically significant enzyme elevation. Mann-Whitney U test of anthropometric changes between participants with and without LEE showed no significant differences in changes in BMI (*U* = 830.5, *P* = 0.75), weight (*U* = 829.0, *P* = 0.74), or waist circumference (*U* = 728.0, *P* = 0.22).

## Discussion

LEE: principal findings and comparison with literature

In this prospective cohort of 101 ART-naïve South Indian PLHIV initiating TLD, 22 (21.78%) participants developed incident LEEs of any grade, including 7 (6.93%) who developed Grade 2 or higher elevations. At the cohort level, mean ALT did not change significantly across the six-month observation period. Participants with Grade 2 or higher LEE showed a spontaneous decline in ALT by six months despite uninterrupted DTG exposure. One participant in the Grade 2 or higher subgroup followed a distinct trajectory with progressively rising ALT through six months; this individual remained asymptomatic and did not develop clinical jaundice or coagulopathy. These observations are consistent with hepatic adaptation, a recognized phenomenon in which the liver develops tolerance to a hepatotoxic insult despite continued exposure through adaptive stress responses and immune tolerance mechanisms [23].

Our any-grade LEE incidence of 22 (21.78%) is broadly consistent with the 15% baseline reported in a recent Indian observational study of DTG-treated PLHIV [14] and with the approximately 7% rate of moderate or severe ALT elevation reported in the large OPERA cohort of 6,102 DTG-treated PLHIV in North America [22], which is closely aligned with our Grade 2 or higher rate of 7 (6.93%). The lower rate (3.2%) reported by Ashta et al. likely reflects differences in baseline hepatic risk, the timing of follow-up visits, or the proportion of switch versus initiation patients [12].

Switch studies provide complementary evidence for DTG's favorable hepatic safety profile: transitioning from older ART (first-generation NNRTIs and boosted PIs) to DTG-based regimens improves liver enzymes in many patients [24,25]. Our findings extend this evidence to the treatment-naïve initiation context: although early transaminase rises occur, they appear largely self-limiting at the cohort level and do not preclude continued therapy.

Longitudinal ALT trajectories and hepatic adaptation

The pattern observed at the cohort level was a transient early rise in mean ALT followed by progressive decline despite continued drug exposure. An identical pattern was observed in six of the seven participants with Grade 2 LEE or higher. A similar finding was reported by Joshi et al., who found that 77% of PLHIV on DTG-based regimens with LEE experienced resolution [14]. These findings may be explained by a phenomenon of hepatic adaptation. Andrade and colleagues have characterized this paradigm as the most likely explanation for why most patients exposed to potentially hepatotoxic drugs do not progress to clinically significant drug-induced liver injury (DILI): the liver mounts a toxic stress response involving immune tolerance and upregulation of detoxification enzymes, which allows for adaptation and resolution of LEE [23]. This framework may explain why most ALT elevations following DTG initiation appear self-limiting. The clinical implication is that empirical drug discontinuation may not be warranted in all cases of LEE, especially in cases with mild elevations and no clinical jaundice, coagulopathy, or symptomatic hepatitis.

While our findings support a model of hepatocellular adaptation characterized by spontaneous resolution of ALT elevations despite continued DTG exposure, they do not exclude the possibility of alternative patterns of liver injury. Emerging evidence suggests that DTG-associated liver injury may also manifest as a predominantly cholestatic process, a phenotype that cannot be adequately assessed using ALT measurements alone.

DTG-associated cholestatic injury

A recent prospective cohort study from southern Ethiopia provides crucial additional context. Abraham et al. reported a 29.1% incidence of DTG-associated DILI in 234 ART recipients, of which 98.5% was cholestatic in pattern - characterized by elevated ALP and bilirubin rather than ALT - with only 1.5% having mixed cholestatic and hepatocellular features [26]. In the Indian pharmacovigilance study, Sekar et al. also reported that ALP elevation was the most common DTG-associated adverse drug reaction (39.0%) [27].

However, the magnitude and temporal characteristics of any DTG-specific cholestatic effect remain uncertain for two reasons. First, existing studies have largely involved ART-experienced or mixed cohorts, making it difficult to distinguish de novo DTG-related cholestatic effects from broader ART-associated ALP elevations. Supporting this, a recent meta-analysis found significantly higher ALP levels among ART-exposed PLHIV compared with HIV-negative controls. In contrast, ART-naïve PLHIV did not demonstrate significant ALP elevation, suggesting that ALP abnormalities are primarily an ART-exposure rather than an HIV-related phenomenon [28]. Both the Abraham et al. and Sekar et al. cohorts included participants with prior ART exposure, limiting the ability to attribute the observed cholestatic signals specifically to DTG [26,27].

Second, neither study reported longitudinal cholestatic biomarker data. As a result, it remains unclear whether DTG-associated ALP elevations are transient and self-limiting or progressive over time. Prospective ART-naïve cohorts with serial assessment of the full liver function panel are needed to address this gap. While our study provides multi-time-point ALT data, the absence of longitudinal ALP and bilirubin measurements means that our findings complement, rather than resolve, the emerging literature on DTG-associated cholestatic injury.

Anthropometric and glycemic trajectories

A notable observation in our cohort was the contrast between the hepatic and metabolic responses to DTG. While the cohort-level mean ALT did not change significantly over six months, body weight (+1.79 kg; *F*(2.02, 201.87) = 12.549, *P* < 0.001) and BMI (+0.71 kg/m²; *F*(2.00, 199.90) = 13.117, *P* < 0.001) rose progressively and monotonically, with significant linear trends and no evidence of plateauing during follow-up. This pattern is consistent with both international and Indian experience. Bourgi et al. reported a mean weight gain of approximately 6 kg over 18 months among treatment-naïve PLHIV initiating DTG-based therapy [9], while Indian studies have reported mean weight gains between 2 and 4 kg over 24-96 weeks, with 16%-27% of participants experiencing ≥10% weight gain from baseline [12,13,15]. Taken together, these findings suggest that clinically meaningful weight gain is a reproducible feature of DTG-based treatment across diverse populations.

Importantly, the trajectory of anthropometric change differed from that of glycemic change. Although RBS levels showed a transient rise during the early months of treatment, values returned to near baseline by six months, and the overall trend did not reach statistical significance. This finding is consistent with Indian observational data suggesting that dysglycemia occurs less frequently and less consistently than weight gain, with reports ranging from minor glucose fluctuations to occasional cases of treatment-emergent diabetes mellitus [13,15]. However, the absence of a sustained glycemic signal in our cohort should be interpreted cautiously. In a large prospective African cohort, Lartey et al. reported incident type 2 diabetes in 11.8% of initially non-diabetic PLHIV within 72 weeks of DTG initiation, with a median time to diagnosis of 24 weeks [10], suggesting that clinically meaningful disturbances in glucose homeostasis may emerge later and require longer follow-up than was available in our study.

Risk factor associations and differential hepatic-metabolic patterns

We did not identify any significant associations between baseline age, sex, changes in weight or BMI, and the development of LEE, consistent with the findings of Joshi et al. [14]. Although limited by sample size, these results suggest that DTG-associated LEE is not readily predicted by routine demographic or anthropometric characteristics and may instead reflect an idiosyncratic hepatic response.

The biological mechanisms underlying DTG-associated weight gain remain incompletely understood. Experimental studies have demonstrated that DTG promotes lipid accumulation, oxidative stress, mitochondrial dysfunction, fibrosis, and insulin resistance in adipocytes, suggesting a direct effect on adipose tissue biology rather than a simple *return-to-health* phenomenon alone [29]. Although our study was not designed to investigate the mechanism, the progressive increase in weight and BMI despite a relatively stable hepatic trajectory is consistent with the concept that DTG-associated metabolic effects may follow a distinct biological pathway from hepatic adaptation. Clinically, these findings emphasize that hepatic and metabolic surveillance address distinct dimensions of treatment safety and cannot be substituted for one another.

Strengths and limitations

This study has several methodological strengths. First, its prospective longitudinal design incorporated five ALT assessments (baseline, one, two, three, and six months), providing sufficient temporal resolution to characterize the evolution of liver enzyme changes following DTG initiation. Second, complete participant retention throughout follow-up minimized attrition and ascertainment bias. Third, the inclusion of only ART-naïve individuals enabled assessment of the de novo hepatic and metabolic effects of TDF/3TC/DTG without confounding from prior antiretroviral exposure or regimen switching. Finally, the concurrent longitudinal evaluation of ALT, anthropometric measures, and glycemic parameters allowed comparison of hepatic and metabolic trajectories within a single cohort receiving a uniform treatment regimen, providing insights infrequently reported in the Indian DTG literature.

Several limitations should be acknowledged. First, only ALT was measured longitudinally, whereas other markers of liver injury, including AST, ALP, gamma-glutamyl transferase (GGT), and bilirubin, were assessed only when clinically indicated. Consequently, isolated cholestatic or mixed-pattern liver injury could not be systematically evaluated, and our findings are restricted to the hepatocellular dimension of DTG-associated hepatic effects. Nevertheless, ALT remains the principal biomarker used for routine hepatic monitoring in Indian ART programs and the most frequently reported marker in studies of DTG-associated LEE. Second, the 6-month follow-up period was sufficient to characterize early ALT trajectories but may not capture late-onset DILI or the longer-term evolution of metabolic changes. The participant with a progressively rising ALT trajectory at six months particularly warrants extended follow-up. Third, although the cohort was adequately powered for the primary outcome of LEE incidence, it was underpowered to detect modest associations with less prevalent risk factors, including alcohol use and concurrent ATT. Fourth, this was a single-center study conducted in a tertiary HIV clinic in South India, which may limit generalizability to other populations and healthcare settings. Fifth, a formal causality assessment using validated tools, such as the Roussel Uclaf Causality Assessment Method (RUCAM), was not undertaken; therefore, observed ALT elevations cannot be attributed exclusively to DTG with complete certainty. Finally, baseline CD4 count was not included as a study variable, limiting our ability to examine its potential influence on the observed hepatic and metabolic trajectories.

## Conclusions

In this prospective cohort of ART-naïve South Indian PLHIV initiated on the TLD regimen, any-grade LEE was relatively common within six months, with Grade 2 or higher elevation being uncommon. The cohort-level mean ALT did not change significantly across the observation period, and most participants with clinically significant elevation showed progressive decline by six months despite uninterrupted therapy, which may be explained by the phenomenon of hepatic adaptation. These findings support the view that mild-to-moderate ALT elevations following DTG initiation can, in most cases, be observed expectantly rather than prompting a regimen change, while emphasizing that close clinical surveillance and, where available, comprehensive liver function monitoring remain essential - particularly to capture cholestatic-pattern injury.

In contrast to the transient hepatic trajectory, body weight and BMI rose progressively and significantly throughout the six-month observation period, shifting the cohort mean BMI from the upper end of the WHO Asia-Pacific normal range into the *overweight-at-risk* category within six months of TLD initiation. These differential trajectories indicate that hepatic and metabolic surveillance address distinct risks and are not interchangeable. As DTG-based therapy continues to scale across South Asia and sub-Saharan Africa, routine monitoring frameworks should retain longitudinal hepatic, anthropometric, and metabolic surveillance to balance the substantial virological advantages of TLD against its potential hepatic and emerging cardiometabolic costs.
